# A machine learning approach for reliable prediction of amino acid interactions and its application in the directed evolution of enantioselective enzymes

**DOI:** 10.1038/s41598-018-35033-y

**Published:** 2018-11-13

**Authors:** Frédéric Cadet, Nicolas Fontaine, Guangyue Li, Joaquin Sanchis, Matthieu Ng Fuk Chong, Rudy Pandjaitan, Iyanar Vetrivel, Bernard Offmann, Manfred T. Reetz

**Affiliations:** 1 PEACCEL, Protein Engineering Accelerator, Paris, France; 2 Department of Chemistry, Philipps-University, 35032 Marburg, Germany; 3 Faculty of Pharmacy and Pharmaceutical Sciences, Monash University, Parkville, Australia; 4 UFIP, UMR 6286 CNRS, UFR Sciences et Techniques, Université de Nantes, Nantes, France; 5 Max-Planck-Institut fuer Kohlenforschung, 45470 Mülheim, Germany

## Abstract

Directed evolution is an important research activity in synthetic biology and biotechnology. Numerous reports describe the application of tedious mutation/screening cycles for the improvement of proteins. Recently, knowledge-based approaches have facilitated the prediction of protein properties and the identification of improved mutants. However, epistatic phenomena constitute an obstacle which can impair the predictions in protein engineering. We present an innovative sequence-activity relationship (innov’SAR) methodology based on digital signal processing combining wet-lab experimentation and computational protein design. In our machine learning approach, a predictive model is developed to find the resulting property of the protein when the *n* single point mutations are permuted (2^*n*^ combinations). The originality of our approach is that only sequence information and the fitness of mutants measured in the wet-lab are needed to build models. We illustrate the application of the approach in the case of improving the enantioselectivity of an epoxide hydrolase from *Aspergillus niger*. *n* = 9 single point mutants of the enzyme were experimentally assessed for their enantioselectivity and used as a learning dataset to build a model. Based on combinations of the 9 single point mutations (2^9^), the enantioselectivity of these 512 variants were predicted, and candidates were experimentally checked: better mutants with higher enantioselectivity were indeed found.

## Introduction

Directed protein evolution is a relatively tedious and time-consuming endeavour. Originally starting from purely random mutagenesis approaches^1,2^, protein engineering has advanced to a more and more information driven effort (site directed, structure based)^3–7^. Directed evolution of stereoselectivity continues to be a central issue of significant importance in organic and medicinal chemistry as well as biotechnology. Such techniques as rationally chosen saturation mutagenesis at sites lining the binding pocket as part of the combinatorial active-site saturation test (CAST)^8^ and iterative saturation mutagenesis using reduced amino acids for minimizing the screening effort^9–13^ constitute important advances. Nevertheless, the screening effort remains the bottleneck of directed evolution of stereo- and regioselectivity, which calls for support and guidance by in silico techniques.

Machine learning algorithms in protein science were developed as early as 1992, in that case for secondary structure prediction^14^. Thereafter new versions of machine learning for predicting structure, folding, binding, and even catalytic activity appeared with the aim of processing the accumulating information about mutants and their properties^15–21^. The “big data” serves as training set for these algorithms to facilitate the prediction of new and improved variants, thereby aiding experimental efforts in protein engineering based on site-specific mutagenesis or directed evolution^6,22^. However, these methods are mainly based on addition of the activities of the characterized single mutations. Hence, the non-additivity of functional mutations can lead to inaccurate identification of the best performing engineered protein using in silico approaches. Epistasis phenomena can impair the predictions in protein engineering and screening. In this sense, machine learning has not been applied in order to evolve enzyme mutants with enhanced or inverted stereoselectivity.

Digital Signal Processing (DSP) techniques are analytic procedures, which decompose and process signals in order to reveal information embedded in them^23^. The signals may be continuous (unending) or discrete such as the protein residues. In proteins, Fourier transform methods have been used for: biosequence (DNA and protein) comparison^24^, characterization of protein families and pattern recognition^25–27^; classification and other structure based studies such as analysis of symmetry and repeating structural units or patterns, prediction of secondary/tertiary structure, prediction of hydrophobic core motifs, conserved domains, prediction of membrane proteins^28–31^, prediction of conserved regions^32^, prediction of protein subcellular location^33^, for the study of the secondary structure content in amino acids sequence^34^ and for the detection of periodicity in protein^35^. More recently new methods for the detection of solenoids domains in protein structures were proposed^36,37^.

Cosić has developed the most known approach using DSP and called Resonant Recognition Model (RRM). Digital Signal Processing techniques have helped analyse protein interactions^26,38^ and made biological functionalities calculable. In these approaches, protein residues are first converted into numerical sequences using one of the available AAindex from this database^39,40^, representing a biochemical property or physicochemical parameter for each amino acid. These numerical sequences are then processed by means of Discrete Fourier Transform (DFT) to present the biological characteristics of the proteins in the form of Informational Spectrum Method (ISM)^41^. ISM procedure has been used to investigate principal arrangement in Calcium binding protein^25^ and Influenza viruses^42,43^. A variant of the ISM, which engages amino acids parameter called Electron-Ion Interaction Potential (EIIP) is referred as Resonant Recognition Model (RRM). In this procedure, biological functionalities are presented as spectral characteristics. This physico-mathematical process is based on the fact that biomolecules with same biological characteristics recognize and bio-attach to themselves when their valence electrons oscillate and then reverberate in an electromagnetic field^26,44^. RRM involves four steps^45^: (i) the conversion of the Protein Residues into Numerical Values of EIIP Parameter; (ii) a zero-padding/up-sampling (iii) the generation of protein spectrum using Fast Fourier Transform (FFT). FFT processed by means DFT to yield Spectral Characteristics (SC) and point-wise multiplied to generate the Cross Spectral (CS) features during the last step. (iv) Cross-Spectral (CS) analysis represents the point-wise multiplication of the Spectral Characteristics (SC). In this approach, a consensus spectrum (defined as a CS of a large group of sequences that share one or more common biological functions) is the final outcome of the method and the starting point for spectral characterization of protein families^46^.

But up to now, the energy spectra obtained after FFT has never been used to go through statistical modelling and predict the effect of mutations on the fitness of an amino acid sequence. The energy spectra have never been used to explore protein sequence-activity or protein sequence/fitness relationship. We propose a new approach based only on the amino acids sequence and using digital signal processing (FFT) and protein spectrum to modelling and predict the biological activity/fitness and for identifying combination of single points amino acid substitutions with improved fitness. There is no report of such method to the best of our knowledge.

Recently, the machine-learning innov’SAR (innovative Sequence-Activity Relationship) methodology appeared in the patent literature, a structure independent mutant library screening approach developed by PEACCEL^47^. It relies on the representation of proteins as spectra based on the physico-chemical properties of the amino acids that constitute the protein. The protein spectrum that comes out from FFT treatment is the starting point.

In the current study, we demonstrate the potential of innov’SAR methodology (a form of artificial intelligence) in efficiently identifying enantioselective mutants of the epoxide hydrolase from *Aspergillus niger* (ANEH). We also investigate innov’SARs’ capacity to predict mutational epistasis and to identify mutants with improved biological activity.

**Figure 1:**
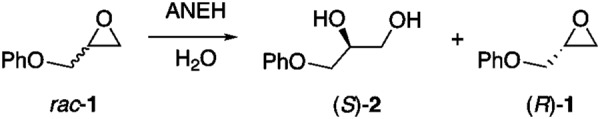
Hydrolytic kinetic resolution of an epoxide (*rac-1*) catalysed by the epoxide hydrolase from *Aspergillus niger* (ANEH).

Epoxide hydrolases (EC 3.3.2.3) are enzymes that catalyse the hydrolytic kinetic resolution of racemic epoxides or the desymmetrisation of meso-epoxides, resulting in the formation of the respective chiral diols in enantiomerically enriched or pure form. Epoxide hydrolases have implications in both the fine chemical and pharmaceutical industries^48^. Sometimes wildtype (WT) epoxide hydrolases are highly stereoselective. In those many cases in which enantioselectivity is poor, directed evolution has been applied successfully, especially using saturation mutagenesis at sites lining the binding pocket (CASTing) and if necessary iterative saturation mutagenesis. For example, the group of Reetz carried out studies on the 398-residue ANEH^49–51^ that is a biocatalyst for the enantioselective hydrolytic kinetic resolution of glycidyl phenyl ether (*rac*-**1**, GPE), (Fig. 1). The WT ANEH has an enantioselectivity factor (E-value) of 4.6, in slight favour of the (*S*)-**2** enantiomer.

**Figure 2:**
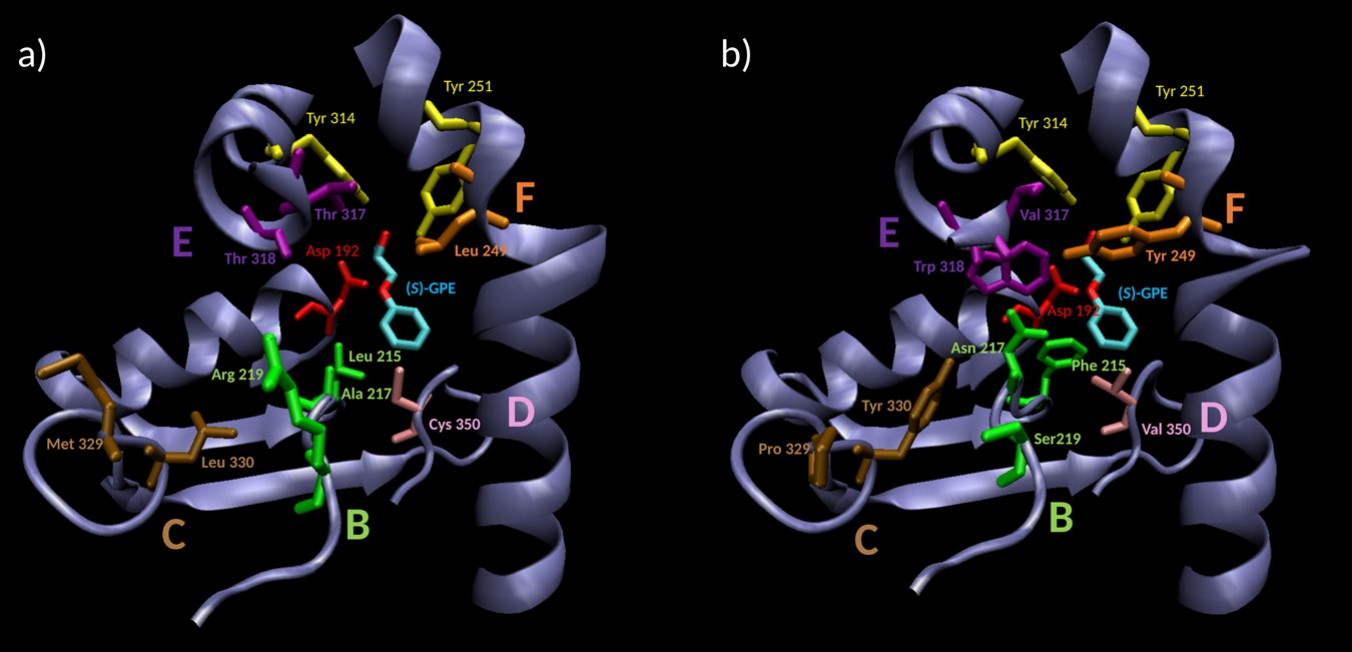
Active site of ANEH. ANEH catalytic triad comprises of Tyr 251 and 314 (in yellow), which orientates the GPE substrate *rac*-*1*, and Asp 192 (in red), responsible for the nucleophilic GPE-ring opening. After a CAST evolutionary process, 9 amino acids from ANEH WT (**a**), gathered in sites B–F, were mutated to yield LW202 (**b**).

In the CAST-study aimed at enhancing the enantioselectivity in the reaction of *rac*-**1**, the ANEH crystal structure served as a guide for identifying randomization sites used in saturation mutagenesis. Five sites (B-F) were chosen for combinatorial randomization using NNK codon degeneracy encoding all 20 canonical amino acids, inducing in each saturation mutagenesis cycle either single, double or triple point mutations (Fig. 2 and Table S1). After 5 iterative rounds of saturation mutagenesis, a total of nine single point mutations led to an improved variant (LW202) with an E-value of 115. Even for this successful approach, this improvement of 25-fold involved at the time a tedious screening effort of around 20000 clones. Although CASTing and iterative saturation mutagenesis (ISM) have since been improved requiring notably less screening^52^, we were interested in exploring the possibility of applying machine learning for efficient directed evolution of enantioselectivity using the same model reaction.

To develop a really useful sequence-based statistical predictor for a biological system, one should observe the following five steps^53^: (i) how to construct or select a valid benchmark dataset to train and test the predictor; (ii) how to formulate the biological sequence samples with an effective mathematical expression that can truly reflect their intrinsic correlation with the target to be predicted; (iii) how to introduce or develop a powerful algorithm (or engine) to operate the prediction; (iv) how to properly perform cross-validation tests to objectively evaluate the anticipated accuracy of the predictor; (v) and as far as possible, how to establish a user-friendly web-server for the predictor that is accessible to the public. Below, we describe how to deal with these steps one-by-one.

## Results

### Modelling approach based on Digital Signal Processing using Fast Fourier Transform (FFT)

innov’SAR requires only, as input data, an initial dataset with the primary sequences of protein variants and their values for a biological activity, to generate a predictive model. This model can be used to find the activity of new mutants, outside of the initial dataset. In contrast to many other rational methods used to find new interesting mutants, innov’SAR can be used without knowledge of the 3D structure. Therefore, innov’SAR can be used for proteins where the crystal structure could not be obtained. 3D structural data could still be used to validate the results from innov’SAR.

innov’SAR consists of 3 phases, namely, the encoding phase, the modelling phase and the predictive phase.

**Figure 3:**
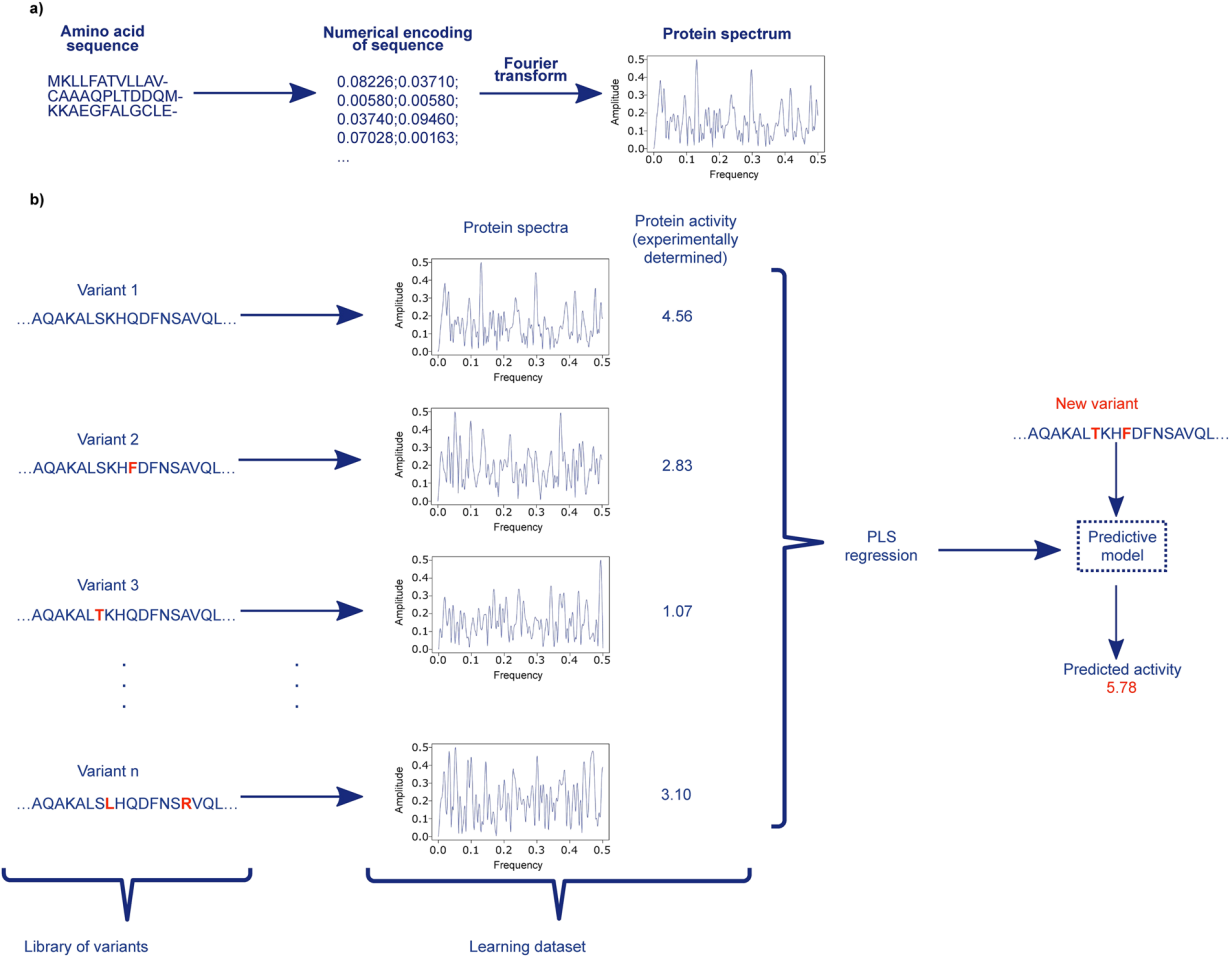
Schematic illustration of innov’SAR methodology. (**a**) A protein sequence is encoded in two steps: i. with a numerical encoding based on an index of AAindex database, i.i. a Fast Fourier transform is applied to convert the encoded sequence into a protein spectrum. (**b**) The different phases of innov’SAR. An encoding phase transforms the primary sequences of the initial dataset into protein spectra. The modelling phase uses the protein spectra and the protein activity as learning dataset in order to construct a regression model. The construction of the model is based on a partial least square regression method, PLS regression, in the modelling of the epoxide hydrolase by innov’SAR. Then the predictive phase uses the regression model and the protein spectra of new variants to have their predicted activity.

In the first phase, the encoding phase, innov’SAR must encode the alphabetic protein sequence into a numerical sequence, understandable by the modelling tools. This phase takes into account only the protein sequences.

innov’SAR uses two steps for the encoding (Fig. 3). First, innov’SAR uses the indexes of the AAindex database [20] to encode the primary protein sequence into a numerical chain, where each letter of amino acid is replaced by a value. This database holds more than 500 numerical indices representing various physicochemical and biochemical properties for the 20 standard amino acids and correlations between these indices are also listed.

The second step comprises a Fast Fourier Transformation (FFT) of the encoded sequences from the first step. FFT is a digital signal processing technique that is used to convert numerical signals into an Energy versus frequency representation (equation 1). After this step, a spectral form of the protein, called the protein spectrum, is generated. The use of FFT and *protein spectra* are the cornerstones for the prediction of biological activity by innov’SAR approach.

1 $$S(k)=\sum _{n=0}^{N-1}s(n){e}^{(-2i\pi {k}_{N}^{n})}$$

With s the input signal (encoded sequence) of length N, S the output spectrum (complex numbers), n the position in the input signal, k the frequency in the spectrum and i the complex number such that i^2^ = −1.

A representation of a protein spectrum is shown in Fig. 3a as a plot *Energy *=* f(frequency)*. The protein spectrum allows to take into account the impact of mutations on the whole spectrum and does not focus on local fitness alone. Therefore, a single point mutation impacts the whole protein spectrum in a similar fashion a single point mutation can impact the whole structure of a protein. At the end of the encoding phase all the variants from the initial dataset will have a protein spectrum.

Only the next phase, the modelling phase, will use the experimental values of the target activity, in conjunction with these protein spectra, in order to identify a predictive model (Fig. 3b). The model is constructed by the application of standard regression approaches based on a learning step and a validation step. innov’SAR used a partial least square regression, PLS, as algorithm of regression to do the model for the predictions of the enantioselectivity of epoxide hydrolase. The protein spectra and the values of the activity are inputs of this regression method for the construction of a model. The goal of the regression method is an attempt to learn and to analyse possible associations between frequencies of protein spectra and the activity values, during the learning step. This learning step leads to the construction of a model. The validation step consists to test the accuracy of the model in order to check if the learning step was efficient. The root mean squared error (RMSE) and the coefficient of determination (R^2^) are the performance parameters to assess a regression model, during the validation step. RMSE values varies between 0 and +∞. R^2^ value varies between 0 and 1. An accurate regression model has an RMSE close to 0 and a R^2^ close to 1.

One particularity of innov’SAR approach, in the modelling phase, is to evaluate multiple encoding indexes to find the best for the construction of models. innov’SAR uses the initial dataset (training set) to construct a predictive model for each encoding index. For each model, innov’SAR calculates the value of the performance parameters in two stages. The first stage is a standard cross validation. The next stage is a modelling integrating the full set in the learning step. The performances from the two stages are analysed to evaluate and to check the robustness and the validity of a model. In the first stage, the cross-validation stage, the initial dataset is split into *k* equal portions. The number *k* varies according to the size of the initial dataset. We use low *k* value if the dataset size is high and high *k* value in the opposite case. We use *k-1* portions as the learning dataset and the remaining one as the test dataset. The procedure is repeated *k* more times until each portion is used as the testing dataset once. The cross-validation allows to avoid potential overfitting problem and to optimize some modelling parameters. The method of cross-validation used for this study is the Leave-One-Out Cross-Validation (LOOCV), where *k* is equal to the number of sequences.

In the second stage, the full set stage, the whole initial dataset is used as a learning dataset and a test dataset will be tested with the optimized parameters from the first stage. This stage checks the accuracy of the predictions for learned sequences.

At the end of the modelling phase, a set of accurate models and their associated encoding indexes are selected and kept.

In the predictive phase, the sequences of the new variants are pre-treated by an encoding phase with a selected encoding index, determined in the modelling phase. Once all the new variants have a protein spectrum, innov’SAR employs a model associated to the encoding index and selected in the modelling phase. Next, the model predicts the values of the activity of the new variants from their protein spectrum (see Fig. 3b).

All steps of the innov’SAR approach were implemented on a workstation equipped with Intel(R) Xeon(R) E5-2650 v4 2.20 GHz processor and 16 GB of RAM. Using this hardware, we were able to handle up to 30 single mutations, generate all combinations of mutations (2^30^) and predict their values of the activity in less than 48 hours. The developed algorithm made use of all of the 12 cores of the CPU to speed up the calculation, but the memory was the main limiting factor to generate and predict the activity of new variants. This methodology can scale to larger machines like High Performance Computing (HPC) clusters to either reduce the computation time or to increase the number of mutations that can be handled at once and ultimately the number of new variants that can be predicted.

### Modelling of the training set of ANEH with innov’SAR methodology

#### Prediction of enantioselectivity with the ΔΔG^‡^ for multiple point mutants using single point mutations or recombinations thereof

Like in most machine learning approaches, innov’SAR needs a training set to learn the correlation between sequence space and experimental values. We wanted to know if it is possible to establish a predictive model with a train set containing only few single point mutations, i.e., without any combinations of mutations allowing to capture epistasis. As noted above, in the original 2006-study, nine single point mutations of ANEH were experimentally evolved and assessed for their impact on the enantioselectivity^49^.

The E-value can be transformed in ΔΔG^‡^ (kcal/mol) by the relation ΔΔG^‡^ = −RT ln (E). We used the ΔΔG^‡^ values for the construction of innov’SAR models, in order to follow and determine the enantioselectivity of the ANEH variants of this study. The energy of the 9-single point mutations and the WT constitute a first learning dataset of protein sequences that was used by innov’SAR and named **dataset A** (Table S2).

A first model, named **model DSA_FFT**, was generated with **dataset A** to predict the ΔΔG^‡^ of double, triple and quadruple point mutants and to appraise the modelling done by innov’SAR with only single point mutants. After the evaluation of multiple encoding indexes by innov’SAR, described previously, the encoding index ZHOH040101^54^ was identified as one of the best to generate an accurate model by innov’SAR and selected to build the **model DSA_FFT**.

**Figure 4:**
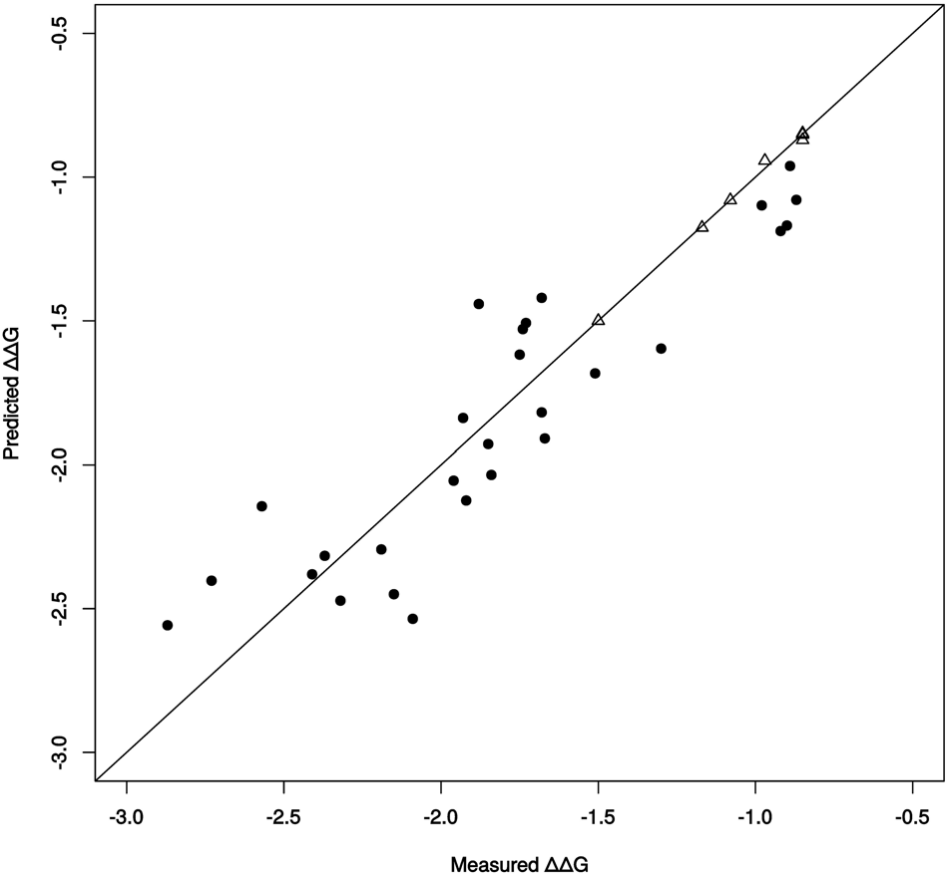
Predictions from model DSA_FFT based solely on experimental values of 9 single point mutants and the WT of ANEH as learning set. The Δ points are the variants for the learning set, the 9 single point mutants and the WT, already learned by the model. The • points are the variants for the validation set, comprising the 28 multiple point mutants, not learned by the model. R^2^ for the learning dataset (Δ): R^2^ = 0.99. R^2^ for the validation dataset (•): R^2^ = 0.81.

The ΔΔG^‡^ values of all possible combinations of these single point mutations, 2^9^ = 512 possible variants, were thus predicted. We created a validation dataset with the 28 multiple point mutants **(**Table S3**)**, including the mutant LW202, that were previously reported (among a total of 512)^50^, in order to check the accuracy of our models based on only 9 single point mutations. The high value of the coefficient of determination, R^2^ = 0.81 from the prediction of the validation dataset, indicates the good accuracy of our model (Fig. 4).

The model uses a learning dataset based only on single point mutations, with relatively high value of ΔΔG^‡^, i.e., low values of enantioselectivity. However, it can determine the multiple point mutants with high experimental values of E-value such as the mutant LW202.

The incorporation of the 28-multiple mutation variants, including the mutant LW202, into the dataset A should improve the accuracy of the model for the prediction of the 512 possible variants. This incorporation formed a new dataset, named **dataset B**, by gathering all the 37 mutants and the WT (Table S4). A new model, named **model DSB_FFT**, was constructed with the **dataset B**. After the evaluation of multiple indices from the AAindex database, the index RACS820104^55^ was used as encoding index due to its higher modelling performance on the new learning dataset.

We compared the performances of the **model DSA_FFT** and the **model DSB_FFT** for the prediction of the 28 multiple point mutants. As the **model DSB_FFT** used already the 28 multiple mutants in the learning step, we performed a Leave-One-Out Cross-Validation (LOOCV) for the validation of the second model. In the LOOCV, the predicted value of each mutant was calculated by removing the mutant from the learning dataset.

**Figure 5:**
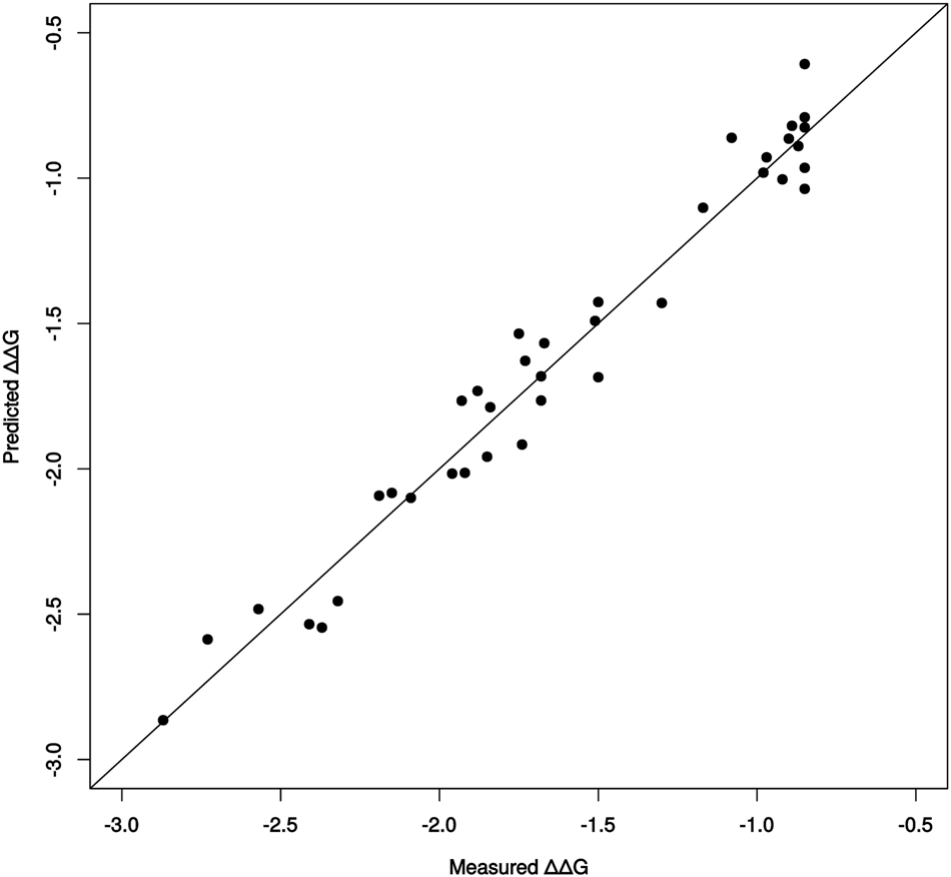
Leave-One-Out Cross-Validation (LOOCV) predictions from model DSB_FFT, based on all the 37 single and multiple point mutants, including LW202 mutant, and the WT of ANEH as learning set. LOOCV performances of prediction: R^2^ = 0.96.

We obtained an excellent R^2^ value (0.96) for the LOOCV of the **model DSB_FFT**, based on the multiple point mutants. Hence, the addition of multiple point mutants to the learning dataset includes some information about the epistasis between mutations and thus improves the accuracy of the prediction model (Fig. 5).

#### Analysis of mutants demonstrating epistatic interactions

It was reported that the recombination of mutations resulted in various non-linear improvements in the desired properties of the mutant ANEH^51^. This cooperativity reflects the effect of epistatic interactions between mutations of ANEH. In a mutant demonstrating epistasis, the association of two or several mutations may result in a different activity than the one resulting from pure addition of each single mutation^56–59^. The effect of combining mutations can be either an addition of mutations where the resulting activity represents the sum of the single activities, or a positive epistasis, with an increase of activity compared to the addition, or a negative epistasis with decreased activity compared to the addition^56–59^.

Hence, it is crucial to determine the capacity for identifying these epistatic interactions of any predictive algorithm, so that mutants with positive epistasis or negative epistasis can be distinguished. We therefore first calculated the theoretical value of ΔΔG^‡^, for the case of pure additivity of mutations, for each of the 28 recombinants, by simply adding the corresponding values of the single mutations (Table S4). The comparison between these theoretical additive values and the measured values allow the evaluation of the impact of epistatic interactions, while revealing whether positive epistasis or negative epistasis is involved. It turned out that all of the 28 variants with multiple mutations have a positive or a negative epistasis: 9 with positive epistasis and 19 with negative epistasis (Table S4).

**Figure 6:**
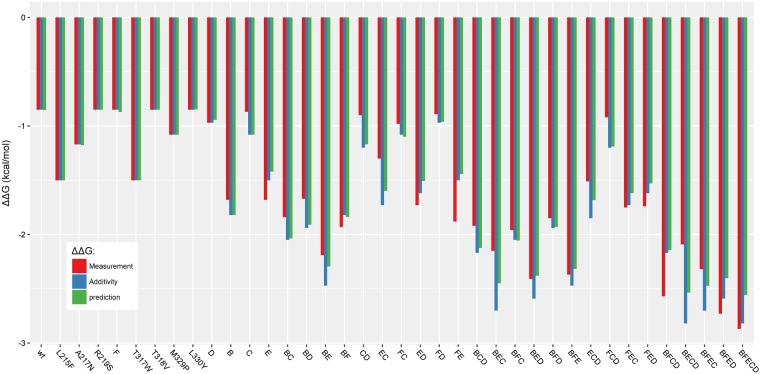
Predictions of ΔΔG^‡^ values for multiple point mutation mutants from model DSA_FFT, based on the WT and the 9 single point mutants. Experimental values are in red, theoretical values for predictions based only on addition of mutations are in blue, and innov’SAR predictions are in green.

After the calculation of the theoretical values, we used them to evaluate the accuracy of predictions based only on the addition of mutations to find the ΔΔG^‡^ of the 28 recombinants. We obtained a R^2^ of 0.80. We already know that the presence of epistasis prevents a perfect accuracy if we consider only the addition of mutations. But for these 28 variants, the predictions based on the addition of mutations provide already a correct accuracy. The **model DSA_FFT**, based on single point mutants, has a performance of predictions (R^2^ = 0.81) close to the performance when the addition of mutations is used. The performances are close, but our model is not based only on the addition of mutations. Indeed, in a next step we compared the predicted ΔΔG^‡^ of the 28 recombinants based on only the addition of mutation and the predicted ΔΔG^‡^ of the **model DSA_FFT** (Fig. 6).

Among the 28 multiple point mutants, the model DSA_FFT gives better results for 18 mutants than the prediction based only on the addition of mutations (Fig. 6 and Table 1). The innov’SAR model allows a distinct capture of epistasis for these mutants, which is a powerful characteristic of this machine learning approach.

**Table 1:** Comparison of the 28 multiple point mutants between the predictions based on addition of mutation and the prediction from the innov’SAR model DSA_FFT and model DSB_FFT.

*Mutant*	model DSA_FFT	model DSB_FFT
*B*	I	I
*C*	I	I
*E*	A	I
*BC*	I	I
*BD*	I	I
*BE*	I	I
*BF*	I	A
*CD*	I	I
*EC*	I	I
*FC*	A	I
*ED*	A	I
*FD*	I	I
*FE*	A	I
*BCD*	I	I
*BEC*	I	I
*BFC*	A	I
*BED*	I	I
*BFD*	I	A
*BFE*	I	A
*ECD*	I	I
*FCD*	I	I
*FEC*	A	A
*FED*	A	A
*BFCD*	A	I
*BECD*	I	I
*BFEC*	I	I
*BFED*	A	A
*BFECD*	A	I

I means better prediction by innov’SAR model and A means better predictions by using only the addition of mutations.

**Figure 7:**
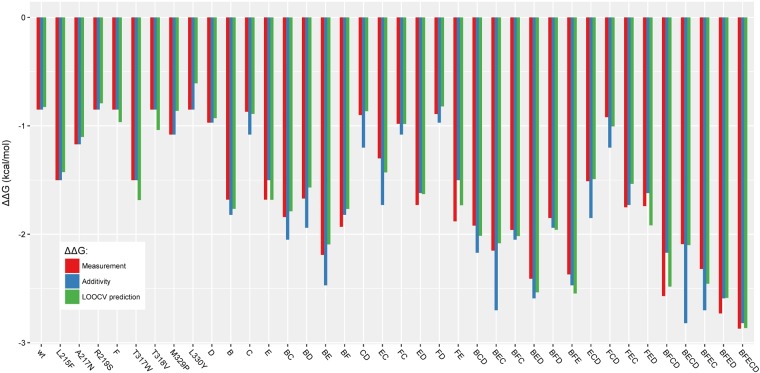
Prediction of ΔΔG^‡^ values by LOOCV method from model DSB_FFT, based on the WT and the 37 single and multiple point mutants. Experimental values are in red, theoretical values for predictions based only on addition of mutations are in blue, and innov’SAR predictions are in green.

We then tested if **model DSB_FFT**, with the incorporation of the 28-multiple point mutants into the 9-single point mutant model could bring improvements to the prediction of epistasis for the other mutants where the **model DSA_FFT** fails to fully capture epistasis. Among the 28 multiple point mutants, the **model DSB_FFT** generates more accurate predictions for 22 mutants, better results than the one based only on the addition of mutations (see Fig. 7 and Table 1). An improvement is observed in the ability of innov’SAR to capture epistatic effects compared to the use of only single mutants in the learning dataset. As already pointed out, this fact is also shown by the higher R^2^ value (0.96) for the LOOCV training set. The LOOCV shows that the model identifies more epistatic effects from the learning dataset.

#### The generation of protein spectra is a key feature of innov’SAR methodology

To get a better idea why innov’SAR approach could predict epistatic effects, we ran different kinds of modelling, with either single and/or multiple mutants and with or without FFT during the encoding phase of innov’SAR (see Fig. 3a).

**Figure 8:**
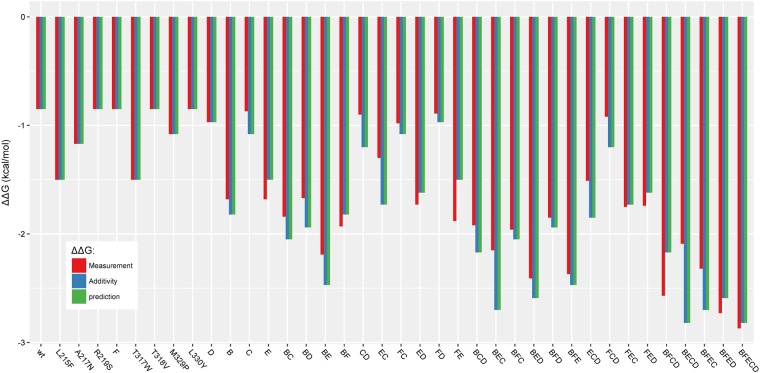
Prediction from model DSA_noFFT, based on WT and 9 single point mutants and without FFT during the encoding phase. Experimental values are in red, theoretical values for predictions based only on addition of mutations are in blue, and innov’SAR predictions without FFT are in green.

Models were built without applying FFT during the encoding phase. When a model based on **dataset A** and without FFT, named **model DSA_noFFT**, the ΔΔG^‡^ predictions perfectly fit with the pure additive values (Fig. 8). In this case, we obtained the same performance as when only the addition of mutations is used to predict the value of ΔΔG^‡^ (R^2^ = 0.80). Without FFT, the **model DSA_noFFT** supposes only the addition of mutation and could not reproduce the epistasis for the prediction.

**Figure 9:**
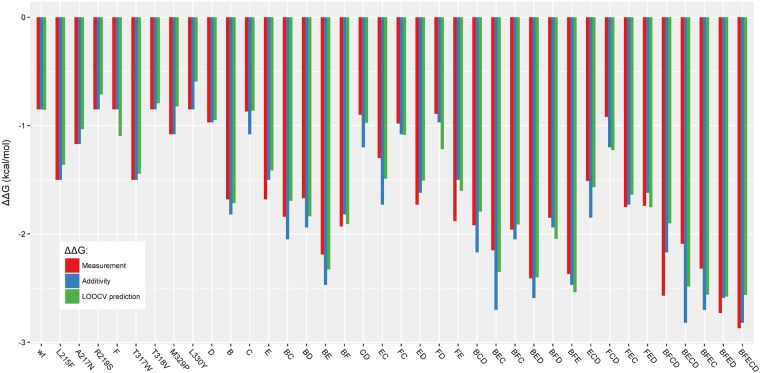
Prediction from model DSB_noFFT, based on the WT and 37 single and multiple point mutants and without FFT during the encoding phase. Experimental values are in red, theoretical values for predictions based only on addition of mutations are in blue, and innov’SAR predictions without FFT are in green.

Next, we then performed the modelling with the dataset B and without FFT to generate the **model DSB_noFFT**. This model has 0.88 as value of R^2^. Figure 9 shows that the **model DSB_noFFT** can predict some epistatic interactions, but with lower accuracy than the **model DSB_FFT**, using FFT in the encoding phase (Fig. 7). Finally, as shown in Fig. 7, a model including FFT improves the accuracy of the predictions of the epistatic interactions of the multiple point mutations. *FFT is an improving factor for the predictions of mutants with epistasis in the models generated by innov’SAR*.

#### Prediction of new improved ANEH mutants

**Figure 10:**
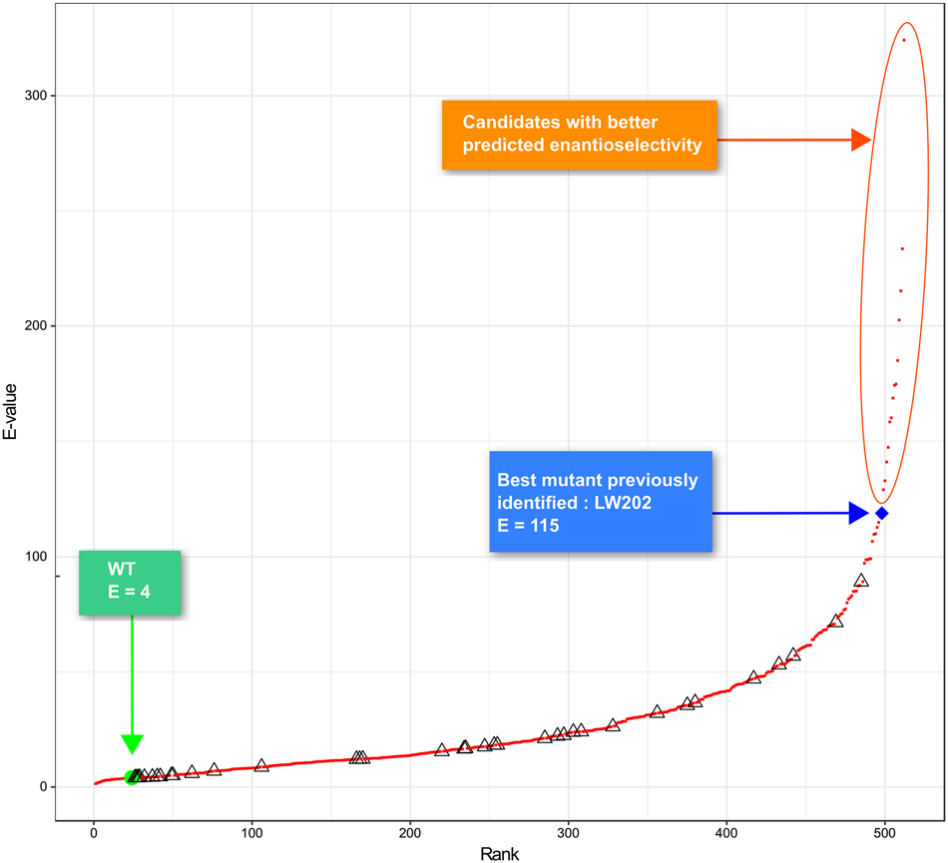
Ranking of the E-values for the 512 possible variants of ANEH with innov’SAR and the model DSB_FFT. (Δ): E-value measured for WT and 37 single and multiple point mutants. (•): E-value predicted for all 512 possible variants.

The best experimental mutant previously described^49^, named LW202, contains all 9 single point mutations in its sequence and has an E-value of 115. In an attempt to identify better mutants, we generated computationally all combinations of the 9 single point mutations (2^9^ variants). This approach resulted in 2^9^–38 = 474 new variants with multiple point mutations. Figure 10 shows the predictions of E-value for all recombinants from the **model DSB_FFT** and our model was able to identify candidates with better predicted enantioselectivity than LW202.

### Experimental characterization of predicted ANEH mutants

In a next step, we made an arbitrary selection of the predicted improved mutants and tested them in wet lab for enantioselectivity. The predicted and experimental values are listed in Table 2.

**Table 2:** Performance of new ANEH mutants as catalysts in the hydrolytic kinetic resolution of *rac-1* as predicted by innov’SAR.

Variant	Mutations	Predicted ΔΔG^‡^	Predicted *E-value*	Experimental *E-value*
WT		−1.07	6	6
P1	A217N_R219S_L249Y	−1.18	7	6
P2	A217N_L249Y_T317W_M329P_L330Y_C350V	−1.98	27	15
P3	L215F_A217N_R219S_L249Y_T317W_T318V_M329P_C350V	−2.86	117	96
P4	L215F_A217N_L249Y_T317W_T318V_M329P_C350V	−3.10	175	253
P5	L215F_A217N_R219S_L249Y_T317W_T318V_L330Y_C350V	−3.14	185	228

**Figure 11:**
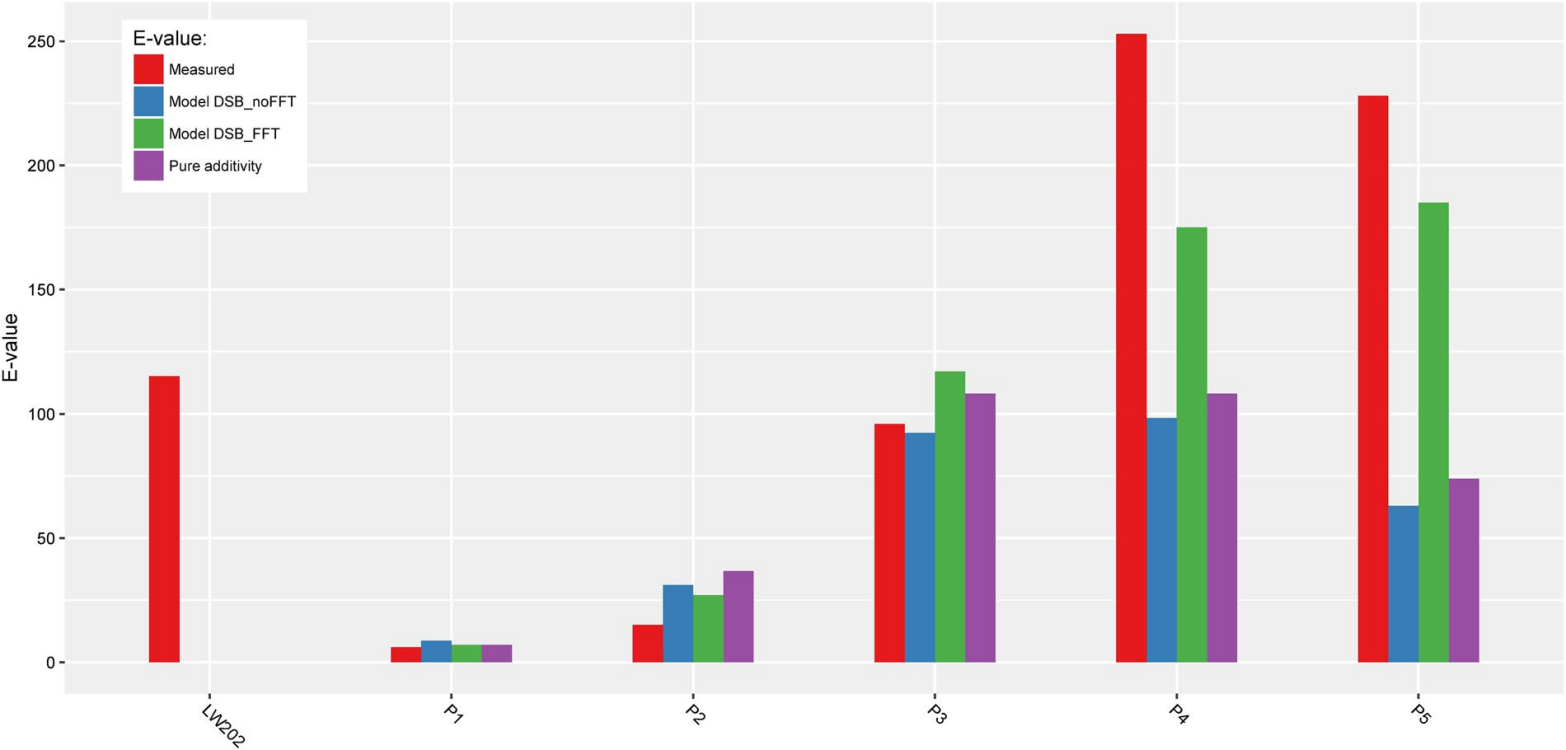
E-values for the 5 mutants identified selected by innov’SAR and for the mutant LW202 originally evolved experimentally. In red: measured E-values, in blue: predicted E-values without FFT during the encoding (model DSB_noFFT), in green: predicted E-values with FFT applied by innov’SAR (model DSB_FFT), in purple: predicted E-value based only on the addition of mutations.

We decided to select 5 mutants comprising a large spectrum of E-values ranging from E-values that are close to the WT and to mutants with E-values that are outside the highest value of the training set. We were able to demonstrate that the 5 mutants have properties that corresponded quite well to the predictions. We also observed that innov’SAR not only allows accurate predictions of E-values that are inside the learning dataset but can also be used to predict and identify superior variants of ANEH. Gratifyingly, a R^2^ value of 0.94 was obtained. With only 37 mutants, our model was able to predict the potential outcome of 512, which means that on the basis of only 7% of the mutants, a whole fitness landscape was predicted. We determined the predictions of E-values of these 5 mutants only when considering the addition of mutations and when no FFT is used with the **model DSB_noFFT** (Fig. 11). We found 0.62 and 0.64 as R^2^ values, respectively, for the predictions based on the addition of mutations and for the predictions generated without FFT. These results show that innov’SAR model performs better for the predictions of these new mutants. It can also be seen that the predicted E-values correspond very well to the experimentally determined enantioselectivity. Especially impressive is the performance of the predicted mutants P4(8) and P5(9), the experimental E-values amounting to 253 and 228, respectively (Table 2). These are distinctly better than mutant LW202 (E = 115) evolved in our original study.

It is important to note that the predictions based on only the addition of mutations cannot find any better mutants than the mutant LW202, because this mutant comprises already all 9 possible mutations and each of these single mutants has an activity equal or better than the WT. The prediction based solely on descriptors without FFT also failed to give predictions of enantioselectivity better than mutant LW202 (Fig. 11). Both models would have therefore not resulted in the identification of improved new recombinants and are not able to predict recombinants which are outside of the training set.

## Discussion

Previously, attempts have been made to study the effect of amino acid substitutions on the activity, function and stability of proteins whose structures have been resolved. This resulted in Quantitative Structure Function Relationship (QSFR) and Quantitative Structure Stability Relationship (QSSR) studies, respectively^60–62^. Particularly, the impact of mutations on the stability of proteins is of specific industrial interest and has been the subject of various studies. Although these structure dependent methods are effective in deriving the correlation between the mutation and its effect on the protein activity, they are limited by their requirement of the availability of the protein structure. The question of enhancing enzyme stereoselectivity was not addressed in these studies.

Hence interest lies in deciphering the impact of mutations irrespective of the availability of structural information, purely based on physico-chemical and other molecular properties of the varying amino acids and statistical analysis thereof. The generation and accumulation of data about protein mutants and their properties as well as the increase of knowledge of protein structure such as enzymes and antibodies has enabled and supported the appearance of knowledge-based predictive algorithms for protein evolution. Quantitative structure-activity relationships (QSAR) have largely been applied to molecule modelling. QSAR methods have also been applied to model peptide or protein activity, but not focused on improving or reversing enantioselectivity^63–65^. It consists in using sets of descriptors derived from sequence information. One of these applications was termed as Protein Sequence Activity Relationship or ProSAR^20^. In this paper, the methodology relied on the binary encoding of the amino acid sequences of the wild type and a collection of few mutants whose activities are known. A statistical model is built to represent the relationship between the mutation and the activity^20^. Subsequent mutant libraries are generated by favouring those mutations that positively affect the activity. The ProSAR method has been demonstrated to show a 4000-fold improvement in the volumetric productivity of the enzyme halohydrin dehalogenase, while maintaining complete (*R*)-selectivity as already shown by the WT^21^. ProSAR was also used to enhance the activity of a transaminase, again with no trade-off in stereoselectivity; in this study, iterative saturation mutagenesis (ISM) at CAST-sites lining the binding pocket was also applied^66^. Both ProSAR and the structure dependent method QSFR, fall under the category of iterative mutant screening methods. The main assumption in iterative mutant library screening methods is that the effects of the mutations are additive in nature^67–69^. The additive nature of the fitness property is exploited mainly to avoid exhaustive and time-consuming search of the vast sequence space. However, in the case of ANEH it was shown that non-additive effects are predominant which makes the application of machine learning methods mainly based on additivity of mutations less efficient. Hence, these methods demonstrate difficulties to predict non-additive epistatic effects as well as long range interactions between amino acids which are non-adjacent to each other. Another method that combines structure activity analysis with machine learning algorithms is the SCHEMA based predictive method developed by the Arnold group^70,71^. Based on a train set of chimeras of the cytochrome P450 enzyme BM3, they developed a linear regression model that allowed the prediction of improved functional chimeras with r = 0.96.

This machine learning method has also been used to predict the properties of membrane bound proteins. Based on SCHEMA, chimeras of channel rhodopsins (ChR) were generated and characterized. The experimental values served as training set for a predictive machine learning application based on regression and Gauss models. Iterative cycles of prediction and empirical testing of ChR chimeras demonstrated that their properties could be efficiently predicted, resulting in the identification of improved variants. In contrast to innov’SAR, this hybrid approach however also requires structural knowledge of the protein^72^.

In the present report, we describe the first application of innov’SAR to guide the choice of mutations to be combined, and to find local or even global optima in the sequence space for enhanced enantioselectivity. A novel statistical model that links protein sequence to protein property was developed and successfully applied. The combination of descriptors and FFT resulted in appropriate predictions and the identification of improved epoxide hydrolase variants in terms of enhanced stereoselectivity. This is mainly due to the capability of the innov’SAR approach to predict the epistasis of interacting mutations. We could demonstrate that the prediction of epistatic effects of combinations of single point mutants is mainly due to the application of the FFT step. This becomes evident when new distinctly improved previously uncharacterized recombinants were correctly predicted, identified and substantiated experimentally. In this case the innov’SAR application comprising FFT was the only method that could predict any improved recombinants, while a pure additive model and innov’SAR without FFT proposed at best only the already known experimental LW202 mutant. This means that these models would have missed the local optima, which is a key criterion for the evaluation of machine learning algorithms.

The difference existing between the predictions generated when only the first step of innov’SAR (which converts amino acids into a series of descriptor dependent numbers) and the FFT (which converts the numbers into a spectrum with different frequencies) suggests that the later plays an important role in the accuracy of the predictions. In addition, we hypothesize that the frequency of a protein spectrum and each amino-acid position in the protein sequence is not linked to one position, but several amino-acid residues or positions at the same time. The frequencies that have a better correlation with the experimental activity contribute better to the prediction of epistatic interactions. We are currently further deciphering the reasons for these observations.

In this work we propose an efficient combinatorial mutant library screening tool for the rational screening and improvement of epoxide hydrolase. While writing this paper, a study appeared describing genetic algorithms to improve an epoxide hydrolases in silico, but stereoselectivity was not treated^73^. Experimental proof of the predictive efficacy of these algorithms was also not provided. In a fundamentally different approach to in silico guidance when evolving the epoxide hydrolase ANEH for enhanced enantioselectivity, we have previously applied the ASRA-algorithm (Adaptive Substituent Reordering Algorithm)^74^. Accordingly, ASRA identifies the underlying regularity of the protein property landscape, in this case enantioselectivity. Consequently, it is not a QSAR-type approach, but a machine learning process very different from innov’SAR.

The present study is the first report that describes the application of innov’SAR in directed enzyme evolution. In this particular case we have limited the application to recombine learned mutations which are contained in the train set. We could identify and experimentally confirmed extremely fit mutants out of 512 possible mutational combinations by using a dataset made of only 7% of the mutants contained in the total fitness landscape. In a next step we are considering going further and to test to what extent our approach is able to predict the properties of non-learned mutations on learned or non-learned positions.

As a conclusion, innov’SAR approach based on sequence information and experimental data combined with Digital Signal Processing such as FFT has demonstrated its ability to capture and predict mutational epistasis. Predictions of new improved versions of the epoxide hydrolase enzyme for enantioselectivity have been confirmed experimentally. This machine learning approach takes into account the interactions between the amino-acids in a protein sequence and is very fast. It opens new opportunities in terms of protein engineering and screening.

As pointed out^75^ and demonstrated in a series of recent publications, user-friendly and publicly accessible web-servers represent the future direction for demonstrating new findings or approaches. Actually, many practically useful web-servers have significantly increased their impacts on medical science, driving medicinal chemistry into an unprecedented revolution^76^, we shall make efforts in our future work to provide a web-server to display the findings that can be manipulated by users according to their needs.

## Materials and Methods

### Materials

KOD Hot Start DNA Polymerase was obtained from Novagen. Restriction enzyme *Dpn* I was bought from NEB. The oligonucleotides were synthesized by Life Technologies. Plasmid preparation kit was ordered from Zymo Research, and PCR purification kit was bought from QIAGEN. DNA sequencing was conducted by GATC Biotech. All commercial chemicals were purchased from Sigma-Aldrich, Tokyo Chemical Industry (TCI) or Alfa Aesar.

### Methods

#### Evaluation of modelling performances by innov’SAR

innov’SAR evaluates the generated models with the values of the root mean squared error (RMSE) and the coefficient of determination (R^2^) during the cross-validation stage and the full set stage. The formulas of these metrics are shown below:

$${R}^{2}=\frac{{({\sum }_{i=1}^{S}({y}_{i}-\bar{y})({\hat{y}}_{i}-\hat{\bar{y}}))}^{2}\,}{{\sum }_{i=1}^{S}{({y}_{i}-\bar{y})}^{2}{\sum }_{i=1}^{S}{({\hat{y}}_{i}-\hat{\bar{y}})}^{2}}$$

$$RMSE=\sqrt{\sum _{i=1}^{S}\frac{{(y-\hat{y})}^{2}}{S}}$$

where, *y*_*i*_ is the measured activity of the i^th^ sequence, *ŷŷ*_*i*_ is the predicted activity of the *i*^th^ sequence, $\bar{y}$ is the average and *S* the number of sequences.

#### PCR based methods for construction of ANEH mutants

The ANEH, constructed in PET-22b^77^, was chosen as template for mutants constructing with over-lap PCR and megaprimer approach^78^. 50 µL reaction mixtures typically contained 30 µL water, 5 µL KOD hot start polymerase buffer (10×), 3 µL 25 mM MgSO_4_, 5 µL 2 mM dNTPs, 2.5 µL DMSO, 0.5 µL (50~100 ng) template DNA, 100 µM primers Mix (0.5 µL each) and 0.5 µL (short fragment PCR) or 1 µL (megaprimer PCR) KOD hot start polymerase. The PCR conditions for short fragment: 95 °C 3 min, (95 °C 30 sec, 56 °C 30 sec, 68 °C 40 sec) × 30 cycles, 68 °C 120 sec. For mega-PCR: 95 °C 3 min, (95 °C 30 sec, 60 °C 30 sec, 68 °C 5 min 30 sec) × 28 cycles, 68 °C 10 min. The PCR products were analysed on agarose gel by electrophoresis and purified using a Qiagen PCR purification kit. 2 µL NEB CutSmart™ Buffer and 2 µL *Dpn* I were added in 50 µL PCR reaction mixture and the digestion was carried out at 37 °C for 7 h. After *Dpn* I digestion, the PCR products 1.5 µL were directly transformed into electro-competent *E. coli* BL21(DE3) to create the final library.

#### Primer design and creation of ANEH mutants

Primer design depend upon the particular amino-acid chosen, and in the case of P1 involves three sites mutation: (1) Amplification of the short fragments of WTANEH using mixed primers P1-A217N/R219S-F/P1-L249Y-R (Table S6); (2) Amplification of the whole plasmid WTANEH using the PCR products of step1 as megaprimers, leading to the final plasmids for mutant P1 generation.

For mutant P2: (1) Amplification of the short fragments of P1 using mixed primers P2-P1-A217N/S219R-F/P2-P1-T317W/M329P/L330Y-R and P2-P1-T317W/M329P/L330Y-F/P2-P1-C350V-R, respectively; (2) Over-lap PCR using the PCR products of step1 as template and mixed primers P2-P1-A217N/S219R-F/P2-P1-C350V-R; (3) Amplification of the whole plasmid of P1 using the over-lap PCR product of step2 as megaprimers, leading to the final plasmids for mutant P2 generation.

For mutant P3: (1) Amplification of the short fragments of P1 using mixed primers P2’-P1-L215F/A217N/R219S-F/P3-P1-T317W/M329P -R; (2) Amplification of the whole plasmid of P1 using the PCR product of step1 as megaprimers, leading to the final plasmid for mutant P2’ generation. (3) Amplification of the short fragments of P2’ using mixed primers P3-P2’-T317W/T318V-F/P3-P2’-C350V-R; (4) Amplification of the whole plasmid of P2’ using the PCR product of step1 as megaprimers, leading to the final plasmids for mutant P3 generation.

For mutants P4 and P5: (1) Amplification of the short fragments of P3 using mixed primers P4-P3-S219R-F/P4-P3-C350V-R and P5-P3-P329M-L330Y-F/P5-P3-R; (2) Amplification of the whole plasmid of P3 using the PCR product of step1 as megaprimers, leading to the final plasmids for mutants P4 and P5 generation. All the primers used are listed in Table S6. The PCR products were digested by *Dpn* I and transformed into electro-competent *E. coli* BL21 (DE3) to create the library.

#### Protein expression

*E. coli* BL21 (DE3) cells carrying the recombinant plasmid were cultivated in 5 mL LB medium containing carbenicillin (100 µg/mL) overnight at 37 °C. The overnight culture was inoculated into 100 mL of TB medium containing carbenicillin (100 µg/mL) and grown at 37 °C. The culture was induced by addition of isopropyl β-D-1-thiogalactopyranoside (IPTG) with a final concentration of 0.2 mM when OD600 reached 0.6, and then allowed to grow for additional 12 h at 25 °C. After centrifugation at 6000 g for 15 min at 4 °C, the bacterial pellet was washed once with phosphate buffer (50 mM, pH 7.4), and resuspended in a phosphate buffer (50 mM, pH 7.4).

#### Hydrolytic kinetic resolution of rac-1

A 1 mL mixture of 50 mM *rac*-1 (10 mM for mutant P2) and recombinant expressed whole cells of WT ANEH or mutants (WT ANEH: OD_600_ = 1, P1: OD_600_ = 0.02, P2: OD_600_ = 25, P3: OD_600_ = 0.02, P4: OD_600_ = 0.3 and P5: OD_600_ = 0.02) in PBS buffer (50 mM, pH 7.4) was stirred at 25 °C. Then 0.2 mL reaction product were extracted with ethyl ether (0.2 mL) at 10 min and 30 min (P2: 1 h, 3 h, 5 h and 7 h), respectively.

## Electronic supplementary material

Supplementary Informations

## Data Availability

All data generated or analysed during this study are included in this published article (and its Supplementary Information files).
