# Publisher Correction: A machine learning approach for reliable prediction of amino acid interactions and its application in the directed evolution of enantioselective enzymes

**DOI:** 10.1038/s41598-021-86884-x

**Published:** 2021-04-12

**Authors:** Frédéric Cadet, Nicolas Fontaine, Guangyue Li, Joaquin Sanchis, Matthieu Ng Fuk Chong, Rudy Pandjaitan, Iyanar Vetrivel, Bernard Offmann, Manfred T. Reetz

**Affiliations:** 1 PEACCEL, Protein Engineering Accelerator, Paris, France; 2 Department of Chemistry, Philipps-University, 35032 Marburg, Germany; 3 Faculty of Pharmacy and Pharmaceutical Sciences, Monash University, Parkville, Australia; 4 UFIP, UMR 6286 CNRS, UFR Sciences et Techniques, Université de Nantes, Nantes, France; 5 Max-Planck-Institut Fuer Kohlenforschung, 45470 Mülheim, Germany

Correction to: *Scientific Reports* https://doi.org/10.1038/s41598-018-35033-y, published online 13 November 2018

The original version of this Article contained a typographical error in Equation 1, where the division line in the fraction “$\frac{n}{N}$” was omitted.

$$S\left( k \right) = \mathop \sum \limits_{n = 0}^{N - 1} s\left( n \right)e^{\big({ - 2i\pi k_{N}^{n} }\big)}$$

now reads:

$$S\left( k \right) = \mathop \sum \limits_{n = 0}^{N - 1} s\left( n \right)e^{{ - 2i\pi k\frac{n}{N}}}$$

The original Article has been corrected.

