# Supplementary material for: A machine learning approach for reliable prediction of amino acid interactions and its application in the directed evolution of enantioselective enzymes
Source: Sci Rep. 2018 Nov 13;8:16757. doi: 10.1038/s41598-018-35033-y (PMC6233173; doi:10.1038/s41598-018-35033-y)
Supplement: Supplementary file 1 — Supplementary Informations [file 41598_2018_35033_MOESM1_ESM.pdf]

# A machine learning approach for reliable prediction of amino acid interactions and its application in the directed evolution of enantioselective enzymes

Frédéric Cadet[1*], Nicolas Fontaine[1], Guangyue Li[2], Joaquin Sanchis[3], Matthieu Ng Fuk Chong[1], Rudy Pandjaitan[1], Iyanar Vetrivel[1], Bernard Offmann[4], Manfred T. Reetz[2,5]

[1] PEACCEL, Protein Engineering Accelerator, Paris, France

[2] Department of Chemistry, Philipps-University, 35032 Marburg, Germany

[3] Faculty of Pharmacy and Pharmaceutical Sciences, Monash University, Parkville, Australia

[4] UFIP, UMR 6286 CNRS, UFR Sciences et Techniques, Université de Nantes, Nantes, France

[5] Max-Planck-Institut fuer Kohlenforschung, 45470 Mülheim, Germany

[*] Corresponding author

Email: frederic.cadet@peaccel.com

## Supporting information

*S1 Table: listing of the sites of mutations found by the CAST method and their composition of mutations*

| Site of mutations | Mutations |
|---|---|
| B | L215F_A217N_R219S |
| C | M329P_L330Y |
| D | C350V |
| E | T317W_T318V |
| F | L249Y |

*S2 Table: the 9 single point mutations and their enantioselectivity measures*

| Variant | E-value | ΔΔG values |
|---|---|---|
| **WT** | 4 | -0.85 |
| **L215F** | 12 | -1.50 |
| **A217N** | 7 | -1.17 |
| **R219S** | 4 | -0.85 |
| **L249Y** | 4 | -0.85 |
| **T317W** | 12 | -1.50 |
| **T318V** | 4 | -0.85 |
| **M329P** | 6 | -1.08 |
| **L330Y** | 4 | -0.85 |
| **C350V** | 5 | -0.97 |

*S3 Table: The 28 multiple point mutants and their enantioselectivity measurements*

| **Variants** | **Mutations** | **E-value** | **ΔΔG values** |
|---|---|---|---|
| **B** | L215F_A217N_R219S | 16.29 | -1.68 |
| **C** | M329P_L330Y | 4.24 | -0.87 |
| **E** | T317W_T318V | 16.29 | -1.68 |
| **BC** | L215F_A217N_R219S_M329P_L330Y | 21.25 | -1.84 |
| **BD** | L215F_A217N_R219S_C350V | 16.02 | -1.67 |
| **BE** | L215F_A217N_R219S_T317W_T318V | 38.01 | -2.19 |
| **BF** | L215F_A217N_R219S_L249Y | 24.68 | -1.93 |
| **CD** | M329P_L330Y_C350V | 4.46 | -0.90 |
| **EC** | T317W_T318V_M329P_L330Y | 8.67 | -1.30 |
| **FC** | L249Y_M329P_L330Y | 5.09 | -0.98 |
| **ED** | T317W_T318V_C350V | 17.70 | -1.73 |
| **FD** | L249Y_C350V | 4.39 | -0.89 |
| **FE** | L249Y_T317W_T318V | 22.71 | -1.88 |
| **BCD** | L215F_A217N_R219S_M329P_L330Y_C350V | 24.27 | -1.92 |
| **BEC** | L215F_A217N_R219S_T317W_T318V_M329P_L330Y | 35.56 | -2.15 |
| **BFC** | L215F_A217N_R219S_L249Y_M329P_L330Y | 25.94 | -1.96 |
| **BED** | L215F_A217N_R219S_T317W_T318V_C350V | 54.77 | -2.41 |
| **BFD** | L215F_A217N_R219S_L249Y_C350V | 21.61 | -1.85 |
| **BFE** | L215F_A217N_R219S_L249Y_T317W_T318V | 51.25 | -2.37 |
| **ECD** | T317W_T318V_M329P_L330Y_C350V | 12.28 | -1.51 |
| **FCD** | L249Y_M329P_L330Y_C350V | 4.61 | -0.92 |
| **FEC** | L249Y_T317W_T318V_M329P_L330Y | 18.30 | -1.75 |
| **FED** | L249Y_T317W_T318V_C350V | 18.00 | -1.74 |
| **BFCD** | L215F_A217N_R219S_L249Y_M329P_L330Y_C350V | 71.45 | -2.57 |
| **BECD** | L215F_A217N_R219S_T317W_T318V_M329P_L330Y_C350V | 32.19 | -2.09 |
| **BFEC** | L215F_A217N_R219S_L249Y_T317W_T318V_M329P_L330Y | 47.17 | -2.32 |
| **BFED** | L215F_A217N_R219S_L249Y_T317W_T318V_C350V | 93.20 | -2.73 |
| **BFECD (LW202)** | L215F_A217N_R219S_L249Y_T317W_T318V_M329P_L330Y_C350V | 117.60 | -2.87 |

*S4 Table: The 28 multiple point mutants and epistasis effects on enantioselectivity*

| **Variants** | **Mutations** | **ΔΔG values** | **ΔΔG from addition of mutations** | **Epistasis** |
|---|---|---|---|---|
| **WT** | | -0.85 | | |
| **L215F** | L215F | -1.50 | | |
| **A217N** | A217N | -1.17 | | |
| **R219S** | R219S | -0.85 | | |
| **L249Y** | L249Y | -0.85 | | |
| **T317W** | T317W | -1.50 | | |
| **T318V** | T318V | -0.85 | | |
| **M329P** | M329P | -1.08 | | |
| **L330Y** | L330Y | -0.85 | | |
| **C350V** | C350V | -0.97 | | |
| **B** | L215F_A217N_R219S | -1.68 | -1.82 | negative |
| **C** | M329P_L330Y | -0.87 | -1.08 | negative |
| **E** | T317W_T318V | -1.68 | -1.5 | Positive |
| **BC** | L215F_A217N_R219S_M329P_L330Y | -1.84 | -2.05 | negative |
| **BD** | L215F_A217N_R219S_C350V | -1.67 | -1.94 | negative |
| **BE** | L215F_A217N_R219S_T317W_T318V | -2.19 | -2.47 | negative |
| **BF** | L215F_A217N_R219S_L249Y | -1.93 | -1.82 | positive |
| **CD** | M329P_L330Y_C350V | -0.90 | -1.2 | negative |
| **EC** | T317W_T318V_M329P_L330Y | -1.30 | -1.73 | negative |
| **FC** | L249Y_M329P_L330Y | -0.98 | -1.08 | negative |

| | | | | |
|---|---|---|---|---|
| **ED** | T317W_T318V_C350V | -1.73 | -1.62 | positive |
| **FD** | L249Y_C350V | -0.89 | -0.97 | negative |
| **FE** | L249Y_T317W_T318V | -1.88 | -1.5 | positive |
| **BCD** | L215F_A217N_R219S_M329P_L330Y_C350V | -1.92 | -2.17 | negative |
| **BEC** | L215F_A217N_R219S_T317W_T318V_M329P_L330Y | -2.15 | -2.7 | negative |
| **BFC** | L215F_A217N_R219S_L249Y_M329P_L330Y | -1.96 | -2.05 | negative |
| **BED** | L215F_A217N_R219S_T317W_T318V_C350V | -2.41 | -2.59 | negative |
| **BFD** | L215F_A217N_R219S_L249Y_C350V | -1.85 | -1.94 | negative |
| **BFE** | L215F_A217N_R219S_L249Y_T317W_T318V | -2.37 | -2.47 | negative |
| **ECD** | T317W_T318V_M329P_L330Y_C350V | -1.51 | -1.85 | negative |
| **FCD** | L249Y_M329P_L330Y_C350V | -0.92 | -1.2 | negative |
| **FEC** | L249Y_T317W_T318V_M329P_L330Y | -1.75 | -1.73 | positive |
| **FED** | L249Y_T317W_T318V_C350V | -1.74 | -1.62 | positive |
| **BFCD** | L215F_A217N_R219S_L249Y_M329P_L330Y_C350V | -2.57 | -2.17 | positive |
| **BECD** | L215F_A217N_R219S_T317W_T318V_M329P_L330Y_C350V | -2.09 | -2.82 | negative |
| **BFEC** | L215F_A217N_R219S_L249Y_T317W_T318V_M329P_L330Y | -2.32 | -2.7 | negative |
| **BFED** | L215F_A217N_R219S_L249Y_T317W_T318V_C350V | -2.73 | -2.59 | positive |
| **BFECD (LW202)** | L215F_A217N_R219S_L249Y_T317W_T318V_M329P_L330Y_C350V | -2.87 | -2.82 | positive |

*S5 Table: Measurement of the new mutants found by iSAR and comparison with the predictions*

| Variant | Mutations | Predicted ΔΔG‡ | Predicted *E-value* | Experimental *E-value* |
|---|---|---|---|---|
| **WT** | | -1.07 | 6 | 6 |
| **P1** | A217N_R219S_L249Y | -1.18 | 7 | 6 |
| **P2** | A217N_L249Y_T317W_M329P_L330Y_C350V | -1.98 | 27 | 15 |
| **P3** | L215F_A217N_R219S_L249Y_T317W_T318V_M329P_C350V | -2.86 | 117 | 96 |
| **P4** | L215F_A217N_L249Y_T317W_T318V_M329P_C350V | -3.10 | 175 | 253 |
| **P5** | L215F_A217N_R219S_L249Y_T317W_T318V_L330Y_C350V | -3.14 | 185 | 228 |

*S6 Table. List of primers for constructing ANEH mutants*

| Name | Sequence (5' to 3') |
|---|---|
| **P1-A217N/R219S-F** | TTTGAACCTGTGC**AAT**ATG**AGC**GCTCCCCCTGAGG |
| **P1-L249Y-R** | CCATGGCATAAGC**ATA**GCCATCGGTCATGA |
| **P2-P1-A217N/S219R-F** | ACCTGTGC**AAT**ATG**AGG**GCTCCCCCTGAGG |
| **P2-P1-T317W/M329P/L330Y-R** | ATAATTCCTTCTG**ATACGG**TGTCGCTCCATTGGGAGCGGAGGCAGTTGGGGT**CCA**CTCGCGGTAGGTAT |
| **P2-P1-T317W/M329P/L330Y-F** | ATACCTACCGCGAG**TGG**ACCCCAACTGCCTCCGCTCCCAATGGAGCGACA**CCGTAT**CAGAAGGAATTAT |
| **P2-P1-C350V-R** | TCCGAGGCACAGG**CAC**AAGGTCCTTGGGG |
| **P2'-P1-L215F/A217N/R219S-F** | GTTCATTTGAAC**TTT**TGC**AAT**ATG**AGC**GCTCCCCCTGAGG |
| **P2'-P1-T317W/M329P -R** | ATAATTCCTTCTGAAG**CGG**TGTCGCTCCATTGGGAGCGGAGGCAGTTGGGGT**CCA**CTCGCGGTAGGTAT |
| **P3-P2'-T317W/T318V-F** | ATACCTACCGCGAG**TGGGTG**CCAACTGCCTCCGC |
| **P3-P2'-C350V-R** | TCCGAGGCACAGG**CAC**AAGGTCCTTGGGG |
| **P4-P3-S219R-F** | AC**TTT**TGC**AAT**ATG**AGG**GCTCCCCCTGAGG |
| **P4-P3-C350V-R** | TCCGAGGCACAGG**CAC**AAGGTCCTTGGGG |
| **P5-P3-P329M-L330Y-F** | CCAATGGAGCGACA**ATGTAT**CAGAAGGAATTAT |
| **P5-P3-R** | AGAATACTAGATTTCCCGTTGTAGCA |

*S7 Table. List of all the iSAR datasets and models used to study the enantioselectivity of ANEH*

| Dataset | Description |
|---|---|
| **Dataset A** | 9 single-point mutants + WT = 10 mutants |
| **Dataset B** | 9 single-point mutants + 27 multiple-point mutants + LW202 + WT =38 mutants |

| Model | Description |
|---|---|
| **DSA_FFT** | Model based on the dataset A with the standard FFT protocol of iSAR for encoding phase |
| **DSA_noFFT** | Model based on the dataset A without FFT applied by iSAR for the encoding phase |
| **DSB_FFT** | Model based on the dataset B with the standard FFT protocol of iSAR for encoding phase |
| **DSB_noFFT** | Model based on the dataset B without FFT applied by iSAR for the encoding phase |
